# Accent processing in dementia

**DOI:** 10.1016/j.neuropsychologia.2012.05.027

**Published:** 2012-07

**Authors:** Julia C. Hailstone, Gerard R. Ridgway, Jonathan W. Bartlett, Johanna C. Goll, Sebastian J. Crutch, Jason D. Warren

**Affiliations:** a Dementia Research Centre, UCL Institute of Neurology, Queen Square, London WC1N 3BG, UK; b Wellcome Trust Centre for Neuroimaging, UCL Institute of Neurology, Queen Square, London WC1N 3BG, UK; c Department of Medical Statistics, London School of Hygiene & Tropical Medicine, London, UK

**Keywords:** Accent, Voice, Frontotemporal lobar degeneration, Alzheimer's disease, Dementia

## Abstract

Accented speech conveys important nonverbal information about the speaker as well as presenting the brain with the problem of decoding a non-canonical auditory signal. The processing of non-native accents has seldom been studied in neurodegenerative disease and its brain basis remains poorly understood. Here we investigated the processing of non-native international and regional accents of English in cohorts of patients with Alzheimer's disease (AD; *n*=20) and progressive nonfluent aphasia (PNFA; *n*=6) in relation to healthy older control subjects (*n*=35). A novel battery was designed to assess accent comprehension and recognition and all subjects had a general neuropsychological assessment. Neuroanatomical associations of accent processing performance were assessed using voxel-based morphometry on MR brain images within the larger AD group. Compared with healthy controls, both the AD and PNFA groups showed deficits of non-native accent recognition and the PNFA group showed reduced comprehension of words spoken in international accents compared with a Southern English accent. At individual subject level deficits were observed more consistently in the PNFA group, and the disease groups showed different patterns of accent comprehension impairment (generally more marked for sentences in AD and for single words in PNFA). Within the AD group, grey matter associations of accent comprehension and recognition were identified in the anterior superior temporal lobe. The findings suggest that accent processing deficits may constitute signatures of neurodegenerative disease with potentially broader implications for understanding how these diseases affect vocal communication under challenging listening conditions.

## Highlights

► Deficits of accent comprehension and recognition occur in common dementia syndromes. ► Alzheimer's and nonfluent aphasia patients show distinct accent processing deficits. ► Cortical substrates of accent processing were identified in anterior temporal lobe.

## 1 Introduction

Communicating with speakers with different accents is an important task that is performed routinely by the healthy brain. Accents signal nonverbal information about speakers, including geographical origins, ethnicity and social milieu. Extraction of this information requires analysis of segmental (phonetic and phonological) speech features and suprasegmental features such as pitch contour, rhythm and stress patterns (Clopper & Pisoni, 2004a,b; De Mareuil & Vieru-Dimulescu, 2006; Evans & Iverson, 2004; Floccia, Goslin, Girard, & Konopczynski, 2006; Howell, Barry, & Vinson, 2006; Van Bezooijen & Gooskens, 1999), and association of these percepts with previously stored knowledge about accents. As an aspect of human meta-linguistic communication, accent processing is likely to bear some similarities to the processing of voice identity (Berman, Mandelkern, Phan, & Zaidel, 2003; Clarke & Garrett, 2004; Clopper & Pisoni, 2004b; Remez, Fellowes, & Rubin, 1997): like voice processing (Belin, Fecteau, & Bedard, 2004; Ellis, Jones, & Mosdell, 1997), the processing of accents is likely to be a computationally demanding, multi-component neural operation recruiting brain mechanisms separable from those encoding the verbal content of speech. In cognitive neuropsychological terms, a word or phoneme spoken in an unfamiliar (foreign or regional) accent has been viewed as an extreme form of native inter-speaker variation (Best, McRoberts, & Goodell, 2001; Clarke & Garrett, 2004; Evans & Iverson, 2004; Floccia et al., 2006; Nathan, Wells, & Donlan, 1998; Schmale & Seidl, 2009) and could be regarded as a ‘non-canonical view’ of that auditory object; a priori, processing non-native accents may engage auditory apperceptive mechanisms analogous to the visual apperceptive mechanisms that process unusual views of visual objects (Goll, Crutch, & Warren, 2010b; Riddoch & Humphreys, 2003; Warrington & James, 1988). The processing of accents therefore generally entails two broadly complementary tasks: processing of the accent as an informative vocal signal in its own right (Adank, Noordzij, & Hagoort, 2012; Berman et al., 2003; Clopper & Pisoni, 2004a,b; Scharinger, Monaham, & Idsardi, 2011; Van Bezooijen & Gooskens, 1999), and processing the effects of the accent on the prototypical speech signal (Adank, Evans, Stuart-Smith, and Scott, 2009; Best et al., 2001; Clarke & Garrett, 2004; Evans & Iverson, 2004; Floccia et al., 2006; Floccia, Butler, Goslin, & Ellis, 2009). The mechanisms that process accents in the healthy brain and the effects of brain damage on accent processing have not been widely studied. Psychophysical studies have demonstrated a perceptual cost associated with comprehension of speech in the presence of an unfamiliar foreign or regional accent (Adank et al., 2009; Best et al., 2001; Clarke & Garrett, 2004; Floccia et al., 2006, 2009); this processing cost has been shown to increase in older adults (Adank & Janse, 2010; Burda, Scherz, Hageman, & Edwards, 2003; Burda et al., 2009), in non-demented aphasic subjects (Burda, Brace, & Hosch, 2007; Burda et al., 2009; Dunton, Bruce, & Newton, 2011) and in patients with Alzheimer's or vascular cognitive impairment (Burda, Hageman, Brousard, & Miller, 2004). Functional imaging evidence has implicated a distributed network including superior temporal gyrus and sulcus, planum temporale, inferior parietal and inferior frontal gyrus in accent processing (Adank et al., 2012; Berman et al., 2003). The components of this network are likely to mediate particular aspects of accent analysis, including vocal timbre (Belin, Zatorre, Lafaille, Ahad, & Pike, 2000; Fecteau, Armony, Joanette, & Belin, 2004), intonation (superior temporal gyrus (STG) and sulcus (STS): (Meyer, Alter, Friederici, Lohmann, & Von Cramon, 2002; Meyer, Steinhauer, Alter, Friederici, & Von Cramon, 2004; Patterson, Uppenkamp, Johnsrude, & Griffiths, 2002; Zhang, Shu, Zhou, Wang, & Li, 2010)) and dynamic phonetic cues (left superior temporal lobe: (Buchsbaum, Hickok, & Humphries, 2001; Chang et al., 2010; Jancke, Wustenberg, Scheich, & Heinze, 2002; Liebenthal, Binder, Spitzer, Possing, & Medler, 2005; Scott, Rosen, Lang, & Wise, 2006; Turkeltaub & Coslett, 2010)). The brain mechanisms involved in accent recognition (association of the accent percept with meaning) are not certain; however, these mechanisms are predicted to engage anterior temporal regions previously implicated in other dimensions of semantic processing, including recognition of voices (Belin & Zatorre, 2003; Nakamura et al., 2001; Von Kriegstein & Giraud, 2004; Warren, Scott, Price, & Griffiths, 2006). The brain organisation of accent processing might be expected a priori to align with other dimensions of person knowledge or with other kinds of geographically differentiated knowledge (Crutch & Warrington, 2003, 2010; Della Rocchetta, Cipolotti, & Warrington, 1998; Ellis, Young, & Critchley, 1989; Gainotti, 2007). However, it is also likely (by analogy with other kinds of complex sound processing) that the perceptual and semantic analysis of accents engages brain mechanisms that are at least partly shared amongst these different cognitive operations, with critical substrates in anterior and lateral temporal cortex (Goll et al., 2010b).

The effects on accent processing of neurodegenerative disease remain largely unknown. However, there are grounds to anticipate deficits of accent processing in the canonical degenerative dementias (Burda et al., 2004). Diseases including Alzheimer's disease (AD) and progressive nonfluent aphasia (PNFA) affect large-scale brain networks including temporal, prefrontal and parietal regions implicated in accent processing (Adank et al., 2012; Berman et al., 2003; Ewers et al., 2011; Gorno-Tempini et al., 2004; Neary et al., 1987; Rohrer et al., 2010; Scahill, Schott, Stevens, Rossor, & Fox, 2002; Seeley, Crawford, Zhou, Miller, & Greicius, 2009; Sonty et al., 2007; Zhou et al., 2010). These neurodegenerative syndromes have overlapping but separable network-level signatures that are likely to reflect an interaction between network properties and the underlying molecular pathology (Seeley et al., 2009; Zhou et al., 2010; Warren, Rohrer, & Hardy, 2012) and which in turn produce characteristic and predictable patterns of neuropsychological deficits. Whereas PNFA predominantly targets an anterior peri-Sylvian network including inferior frontal, insular and superior temporal cortices, AD predominantly targets a more posterior temporo-parietal network extending from mesial temporal lobes to the temporo-parietal junction and medial and lateral parietal cortices (Seeley et al., 2009; Zhou et al., 2010). These network signatures provide an anatomical substrate for the distinctive profiles of auditory cognitive deficits observed in these syndromes (Goll et al., 2010a,b, 2011). Impaired processing of complex nonverbal auditory patterns (Baird & Samson, 2009; Eustache et al., 1995; Goll et al., 2010a, 2011, 2012; Rapcsak, Kentros, & Rubens, 1989; Uttner et al., 2006) and other meta-linguistic components of the speech signal, including prosody (Allender & Kaszniak, 1989; Horley, Reid, & Burnham, 2010; Rohrer, Sauter, Scott, Rossor, & Warren, 2012; Taylor & Warrington, 1971; Testa, Beatty, Gleason, Orbelo, & Ross, 2001) and speaker identity (Hailstone et al., 2011) have been documented in AD and PNFA. However, whereas AD has been associated with more severe deficits of apperceptive auditory processing (for example, identification of sounds under altered listening conditions), PNFA has been associated with more severe deficits of auditory object (timbral) encoding and sound recognition (Gates, Beiser, Rees, D'Agostino, & Wolf, 2002; Goll et al., 2010a,b, 2011; Hailstone et al., 2011). Distinct neuroanatomical associations have been identified for these different patterns of central auditory impairment: anterior temporal cortices have been previously implicated in identity and emotion processing in voices and more generally, in processing extended auditory patterns (such as those embodied in music) (Ellis et al., 1989; Joubert et al., 2006; Rosen et al., 2006; Gainotti, Ferraccioli, Quaranta, & Marra, 2008; Hailstone, Crutch, Vestergaard, Patterson, & Warren, 2010; Hailstone et al., 2011; Omar, Hailstone, Warren, Crutch, & Warren, 2010a; Omar, Rohrer, Hailstone, & Warren, 2010b; Hsieh, Hornberger, Piguet, & Hodges, 2011); while more posterior superior temporal cortices have been implicated in the analysis of sound sources (such as voices) in changing auditory environments (Goll et al., 2012). Damage involving anterior or more posterior temporal cortical regions might lead to deficits in decoding a range of complex auditory signals, including accents: profiles of impairment in different neurodegenerative diseases are likely a priori to be partially separable, but temporal cortex may contain critical brain substrates that are common to different diseases and different levels of accent processing (Goll et al., 2010b).

In this study we set out to investigate the cognitive and neuroanatomical bases of accent processing in two canonical neurodegenerative dementia syndromes: typical amnestic AD and PNFA. We designed a novel neuropsychological battery to assess these cognitively impaired patients, addressing two aspects of accent processing: the intelligibility of accented speech (accent comprehension) and recognition of non-native regional and international accents (accent recognition). Neuroanatomical associations of behavioural performance within the larger AD group were assessed using voxel based morphometry (VBM). Based on previous evidence with other aspects of complex sound processing (Goll et al., 2010a,b, 2011; Hailstone et al., 2011), we hypothesised that patients with AD would have particular difficulty in tracking auditory information (e.g., comprehending spoken sentences) presented under less familiar accents, since this is likely to depend on accurate apperceptive processing; whereas patients with PNFA would have particular difficulty with accent decoding (e.g., comprehending single words) since this is likely to depend on accurate representation of auditory object properties in phonemes and syllables. We hypothesised that both patient groups would show impairments of accent recognition, since this is likely to be in part contingent on accurate perceptual encoding, as well as additional, potentially vulnerable semantic mechanisms (Goll et al., 2010b). We further hypothesised that accent comprehension and accent recognition performance would have neuroanatomical associations in postero-lateral and anterior temporal lobe cortical regions previously shown to be critical for other aspects of vocal signal processing.

## 2 Materials and methods

### 2.1 Subject details

Twenty patients with AD and six patients with PNFA diagnosed according to consensus clinical criteria (Dubois et al., 2007; Neary et al., 1998) were recruited via the tertiary Cognitive Disorders Clinic at the National Hospital for Neurology and Neurosurgery. Patients with AD recruited to this study all had a typical clinical history of memory-led cognitive decline, rather than one of the AD variant presentations. Thirty five healthy older control subjects with no history of neurological or psychiatric illness also participated. The study was approved by the local institutional research ethics committee and all subjects gave informed consent in accord with the principles of the Declaration of Helsinki.

All subjects were native British residents with English as their first language. In order to gather information about their past accent exposure, all subjects completed a questionnaire detailing the region of Britain where they had grown up, their recent regional residence and any extended periods (>6 months) spent outside the United Kingdom. This information indicated that the overall accent exposure of the subject groups was likely to have been similar. All subjects were resident in Southern England at the time of participation in the study and most had grown up in South-Eastern England. One patient with PNFA had spent her early childhood abroad (Malta) and one patient with PNFA, six patients with AD and nine healthy control subjects had spent their childhood in another region of Britain; nine patients with AD and ten healthy control subjects had lived outside the United Kingdom for an extended period (most during their earlier adult life).

Demographic and clinical details of subjects are summarised in Table 1. Background data for the healthy control and AD groups were included in a previous study (Hailstone et al., 2011). Patient and control groups were well-matched for age. Fisher's exact test was used to assess group differences in gender, for all other variables differences in means were assessed using *z*-tests with bootstrap (2000 replicates) standard errors. Males were under-represented in the PNFA group relative to AD and control groups (although these differences were not statistically significant), and controls had a significantly greater average number of years of education compared to both patient groups (the patient groups did not differ significantly on this measure). In order to take account of any group-wise effects on accent processing from age, gender or years of education, these factors were included as nuisance covariates in all analyses of behavioural data, as described below. The AD and PNFA groups did not differ significantly on one measure of disease severity (Mini Mental State Examination score) but the AD group had a significantly longer mean symptom duration than the PNFA group.

Eighteen patients in the AD group and all patients in the PNFA group had undergone previous brain MRI; these images were reviewed by an experienced neuroradiologist. Fifteen of the AD patients had disproportionate bilateral hippocampal atrophy and the remainder had generalised cerebral atrophy. Two AD patients were unable to have MRI due to a cardiac pacemaker; computed tomography in one of these patients showed generalised cerebral atrophy. Patients with PNFA showed bilateral but asymmetric peri-Sylvian atrophy (more marked on the left in three cases and on the right in the remaining cases). No patient had radiological evidence of significant cerebrovascular disease.

### 2.2 General neuropsychological assessment

All patients and 19 healthy control subjects had a comprehensive general neuropsychological assessment; 16 control subjects performed a reduced set of tests. The tests administered are listed in Table 2. Groups were compared using linear regression, adjusting for age, gender, and years of education, with *p*-values from *z*-tests using bootstrap standard errors (2000 replicates).

### 2.3 Assessment of peripheral hearing

Most subjects had no clinical history of hearing loss. One AD patient had mild bilateral high frequency hearing loss. One PNFA subject had bilateral high frequency hearing loss, assessed on clinical audiometry. One control subject had mild bilateral high frequency hearing loss, previously confirmed on clinical audiometry. To assess any effects of peripheral hearing loss on performance in the experimental tasks across the experimental groups, all subjects underwent screening pure tone audiometry. Tones were administered via headphones from a notebook computer in a quiet room. The procedure was adapted from a commercial screening audiometry software package (AUDIO-CD^TM^, http://www.digital-recordings.com/audiocd/audio.html). Five frequency levels (0.5, 1, 2, 3, 4 KHz) were assessed and at each frequency, subjects were presented with a continuous tone that slowly and linearly increased in intensity (1 dB/s). Subjects were instructed to tap as soon as they could detect the tone and the response time was stored for offline analysis. The mean value of response time (i.e., detection threshold) for three presentations of the same tone in the right ear was taken as the detection threshold for that frequency; four subjects (one AD patient, two controls) reported unilateral right sided hearing loss and in these subjects the left ear was tested. Group differences in mean response time at each frequency were assessed using linear regression adjusted for age and gender, with *p*-values from *z*-tests using bootstrap standard errors (2000 replicates).

### 2.4 Experimental investigations: Plan and general procedure

Accent comprehension was assessed in two tasks: comprehension of questions spoken in a native British (Southern English) versus an international accent of English, and verification of single words spoken in an international accent relative to the native British English accent. These tests were designed to assess patients' ability to perceptually encode accent characteristics. In both tests, the speech signal must be comprehended under non-canonical listening conditions; we reasoned that sentence comprehension would be more likely to depend on accurate representation of suprasegmental accent characteristics, while word verification would particularly engage segmental processing mechanisms. Sentence comprehension under less familiar accents entails ‘apperceptive’ processing of a series of non-canonical ‘views’ of the speech signal; we argue that word verification is likely to be relatively more dependent on accurate encoding of individual speech sounds according to their characteristics as auditory objects. We use ‘Southern English’ here to designate an English accent comprising features of the so-called ‘Home Counties’ or ‘Estuary’ regional accents; this accent was adopted as the reference accent because it is widely used in broadcasting, particularly common in the Greater London area, and likely to be highly familiar to most participants.

Accent recognition (semantic processing of accent characteristics) was assessed in three tasks, directed to progressively more fine-grained ‘levels’ of accent knowledge: identification of an accent as British or international English, identification of regional British accents and identification of regional English accents.

For both the accent comprehension and accent recognition limbs of the battery, additional tests were administered to assess other cognitive capacities relevant to performance on the accent tasks: these additional tests comprised Southern English phoneme discrimination (a measure of phonological processing, relevant to performance on the word verification subtest) and country recognition (a measure of general geographical semantic knowledge, relevant to performance on the accent recognition subtests).

Auditory stimuli were presented from digital wavefiles via headphones at a fixed comfortable listening level in a quiet room. The tests were presented in a fixed order to all participants; within a test, the order of stimuli was randomised. Before beginning each test, several practice trials were administered to ensure the subject understood the task. Stimuli were each presented once only and no feedback was given about performance during the test. No time limit was imposed. The experimental tests were administered to subjects over several sessions.

#### 2.4.1 Accent comprehension

Accent comprehensibility is influenced by lexical context, familiarity (Adank et al., 2009; Clopper & Bradlow, 2008) and acoustic (phonological-phonotactic) distance from native speech (Best et al., 2001; Clarke & Garrett, 2004; Floccia et al., 2006); in these respects, other regional accents generally fall closer to native accent than do foreign accents. For this subtest, three international accents were chosen which differ from Southern English at the phonological-segmental and prosodic level: General American, South African and Australian. We hypothesised that British subjects would be more familiar with American and Australian accents (via the media) than with the South African accent, though we anticipated that subjects would have been fairly familiar with all the international accents selected for this study. All recorded speakers had English as their first language (in order to eliminate any perceptual costs associated with irregular or dysfluent speech of non-English speakers (Floccia et al., 2006)) and all were female aged 20–43 years; international accent speakers had all lived in the United Kingdom for less than nine months (speaker characteristics are summarised in Supplementary Table S1 on-line). To minimise any effects from individual speech idiosyncrasies and to increase variation in speaker identity across the stimulus set, more than one speaker was recorded for each accent (four Southern English, two American from the Mid-West and California, two South African from Johannesburg and two from Eastern Australia). Speech samples were recorded as digital wavefiles (sample rate 44.1 kHz) using an external microphone (Samson^®^) C01U USB Studio Condenser) onto a notebook computer running Audacity^®^ software. Examples of the stimuli are available from the authors.

**Question comprehension.** In this subtest, subjects heard 40 short spoken questions (each between four and eight words in length) designed to elicit a one word answer (Prof EK Warrington, unpublished; see Supplementary Table S2 on-line); each sentence was spoken once in a Southern English accent and once in an international accent (either American or South African), yielding 80 trials in total (40 trials for the English accent and 40 trials for a ‘foreign’ accent). Trials were presented in four divided blocks of 20 trials. For each sentence, the presentation order was randomly assigned first to the English accent or first to an international accent in the first set of 40 trials and assigned to the other accent category (using a different randomisation order) in the second set of 40 trials. The task on each trial was to answer the question (either aloud, or in the case of patients with PNFA, as a written response if preferred).

**Word verification.** In this subtest, subjects heard 24 spoken monosyllabic words derived from PALPA (Psycholinguistic Assessment of Language Processing in Aphasia) Minimal Pair Discrimination tests (Kay, Lesser, & Coltheart, 1992) (see Supplementary Table S3 on-line) each recorded with Southern English, American, Australian and South African accents. Each spoken word was presented twice using each accent (yielding 48 trials for each accent and 192 trials in total), once with the target written word and once with a foil. Stimuli were presented using Superlab version 4 (http://www.superlab.com/). On each trial the subject was instructed to read a written word (target or foil, with equal probability) presented on a computer screen; the subject then heard the spoken word, and the task was to indicate whether this spoken word matched the written word.

Word foils each contained a single phonetic change compared with the corresponding target word (half contained a change in vowel sound, half contained a change in initial or terminal consonant). The set of words contained a range of vowel and consonant changes; no attempt was made to manipulate confusability under particular accents. In order to enhance any effect of accent on error rates (and/or reaction times in controls) target words selected had an orthographic and phonological neighbourhood >10 (Grainger, 1990; Luce & Pisoni, 1998) and foils had a CELEX word frequency (Baayen, Piepenbrock, & Gulikers, 1995) greater than the corresponding target word. ‘Neighbourhood’ here refers to the number of similar words of the same length generated by changing one letter while preserving letter position; increasing neighbourhood size is associated with increasing lexical decision time. Psycholinguistic statistics used in this study were obtained using N-Watch http://www.maccs.mq.edu.au/∼colin/N-Watch/.

Trials were presented in eight blocks each containing the set of 24 spoken words (six words spoken with each of the accents); the presentation order of a particular word under each accent was randomised. Patients responded by pointing to ‘Yes’ or ‘No’ listed for each trial in a response sheet; control subjects responded by pressing ‘Yes’ or ‘No’ on a response box, and their reaction times were also recorded. As no predictions were made about how confusable distractor words were for each accent, only trials in which the target written word matched the spoken word were analysed, yielding a score/24 for each accent.

**Phoneme discrimination.** As a measure of phonological processing ability, a total word verification score was calculated for all target words and foils spoken with a Southern English accent (total score/48). Words and foils were derived from the PALPA Minimal Pairs Discrimination tests (Kay et al., 1992) (see Supplementary Table S3 on-line); each ‘target’ word differed from the corresponding ‘foil’ word with respect to one phonetic feature. The task on each trial was to indicate whether the spoken word matched the written word. Patients responded by pointing to ‘Yes’ or ‘No’ listed for each trial in a response sheet; control subjects responded by pressing ‘Yes’ or ‘No’ on a response box.

#### 2.4.2 Accent recognition

**International accents.** In this subtest, subjects were assessed for their ability to identify an accent as native British (Southern English) or international. The same set of 40 questions and accents (Southern English, American, South African) used in the question comprehension subtest was re-presented. Maps were used to assist in explaining the task. On each trial the subject was asked ‘Is this person from England?’, and responded ‘Yes’ or ‘No’ verbally or by pointing on a response sheet. If the subject scored <12 on the first block of 20 trials the test was discontinued; scores for the first block and for all four blocks were analysed.

**Regional British accents.** For this subtest, audio samples each comprising 7–15 s of speech representing a Southern English, Irish, Scots or Welsh accent were obtained from accent archives available on the World Wide Web (http://web.ku.edu/∼idea/; http://www.bbc.co.uk/voices/; http://www.bl.uk/learning/langlit/sounds/index.htm; stimuli are listed in Supplementary Table S4). In selecting the clips, we attempted to minimise extraneous lexical cues to accent origin. Six different speakers representing each of the four accents were selected, yielding a total of 24 trials. The task on each trial was to identify the speaker's regional origin in a four-alternative forced choice procedure (England, Ireland, Scotland, Wales); a map of the United Kingdom and Ireland labelling each region was also presented with which to respond non-verbally if preferred.

**Regional English accents.** This subtest was designed to exploit the wide variation in English regional accents as an index of more fine-grained semantic processing of accents. Audio samples representing speakers from either the north or the south of England were selected from the on-line accent archive (http://web.ku.edu/∼idea/; http://www.bbc.co.uk/voices/; http://www.bl.uk/learning/langlit/sounds/index.htm; stimuli are listed in Supplementary Table S4), following the same selection criteria as the regional British accents subtest. 24 audio clips each representing a different speaker from either the north or the south of England were chosen (avoiding the Midlands, in order to reduce ambiguity), yielding a total of 24 trials. The task on each trial was to identify the speaker's regional origin in a two-alternative forced choice procedure (North or South England); a map of England labelling each region was also presented with which to respond non-verbally if preferred.

**Country knowledge.** As a measure of general geographical knowledge, knowledge of 10 countries (four British, four European and two non-European; see Supplementary Table S5 on-line) was assessed in three subtests: naming from verbal description; naming from maps; and (if the subject was unable to name all 10 countries) recognition of the map corresponding to the spoken name of the country (forced-choice from an array of 10 maps).

### 2.5 Analysis of behavioural data

Behavioural data were analysed in STATA release 9.2 (Stata Corporation, College Station, TX). To quantify differences between groups (control, AD, PNFA) on each experimental test, linear regression models were fitted to the scores, including group membership as a covariate and also adjusting for age, gender and years of education. *P*-values for group differences were found using a *z*-test with bootstrap standard errors (2000 bootstrap replicates). In order to investigate the performance cost associated with listening to words or sentences presented in an international accent on accent comprehension tests, a difference score (mean score for international accents minus total score for Southern English) was calculated for each subject based on their performance on the question comprehension and word verification subtests. Differences between groups for these scores were again assessed using linear regression, adjusting for age, gender and education.

In addition, on the word verification test, to investigate differences in score by accent (Southern English, American, Australian, and South African) in the healthy control group, differences in mean score between each accent and English were calculated and 95% Wald-type bootstrap confidence intervals (2000 replicates) were obtained. A linear regression model was used to estimate differences in mean reaction time between accents, adjusting for word duration. *P*-values and 95% confidence intervals were again found using *z*-tests and Wald intervals using bootstrap standard errors (2000 bootstrap replicates).

### 2.6 Neuroimaging data

**Brain image acquisition.** For 17 AD patients, T1-weighted volumetric MR brain images were acquired at the time of behavioural assessment on a Siemens Trio TIM 3T scanner (Siemens Medical Systems, Erlangen, Germany) using a 3D magnetization-prepared rapid gradient echo (MP-RAGE) sequence. 208 sagittal partitions of 1.1mm thickness were acquired with 28-cm field of view and a 256×256 acquisition matrix, producing 1.1 mm isotropic voxels.

**VBM analysis.** Brain images were processed using MATLAB 7.2 (The MathWorks, Inc., Natick, MA, USA) and SPM8 software (Statistical Parametric Mapping, Version 8; http://www.fil.ion.ucl.ac.uk/spm) with default settings for all parameters; normalisation was performed using the DARTEL toolbox (Ashburner, 2007).

Raw brain images were manually rigidly reoriented to standard space (the international consortium for brain mapping (ICBM) template) and the reoriented scans were segmented into grey and white matter using the new Segmentation Toolbox in SPM8. “Imported” grey and white matter segmentations were used in the DARTEL toolbox (Ashburner, 2007) which iteratively registered the segments to an evolving estimate of their group-wise average (Ashburner & Friston, 2009). The grey matter segments were normalised using the final DARTEL transformations and modulated to account for local volume changes (Ashburner & Friston, 2000). Finally, images were smoothed with a Gaussian kernel (6 mm full-width-at-half-maximum).

Our prior anatomical hypothesis concerning the existence of critical temporal lobe substrates for accent processing motivated us to construct anatomical small volumes for assessing regionally-specific anatomical associations of performance in the behavioural tasks. These temporal lobe volumes were created manually in MRIcron^®^ (http://www.cabiatl.com/mricro/mricron/index.html) from a study-specific template generated by warping all native space whole-brain images to the final DARTEL template and calculating the average of the warped brain images. Separate small volumes were created for the right and left temporal lobes. Each regional volume was intentionally generous to ensure adequate coverage of the whole temporal lobe, extending from the pole to the temporo-parietal and temporo-occipital junctions. All attributions within each small volume were subsequently inspected to ensure anatomical accuracy.

Within the AD group, associations with regional grey matter volume were assessed separately for performance on each of the accent comprehension subtests (entering the difference score for each subtest) and on each of the accent recognition subtests (entering the raw score for each subtest). For each experimental subtest, grey matter volume was modelled as a function of the experimental test score and subject age and total intracranial volume (TIV) were included as covariates; TIV was measured outside SPM using a previously described procedure (Whitwell, Crum, Watt, & Fox, 2001).

An explicit analysis mask was used to exclude any voxels for which more than 20% of individual images had an intensity value <0.1 (this proportional thresholding procedure has been shown to improve visualisation of markedly atrophic brain regions compared with the default “absolute thresholding” mask option in SPM (Ridgway et al., 2009)). For each test, grey matter associations were assessed over the whole-brain and within the temporal lobe regional volume of interest specified by our prior anatomical hypotheses; a voxel-wise statistical threshold *p*<0.05 family-wise-error (FWE)-corrected for multiple comparisons was applied in all analyses. Statistical parametric maps were displayed as overlays on the group mean template structural brain image. The grey matter segment of the final DARTEL template was affine registered to the a priori grey matter tissue probability map in SPM, and DARTEL coordinates were transformed using the estimated affine mapping to standard stereotactic MNI space; MNI coordinates of local maxima are displayed in Table 4.

## 3 Results

### 3.1 General neuropsychological performance

Relative to healthy controls, both patient groups showed significant impairment on tests of IQ, verbal recognition memory, semantic memory tests, working memory, arithmetic and executive function (see Table 2) after adjusting for age, gender and number of years of education. In addition, the AD group was impaired relative to controls on tests of face recognition memory and object perception. The PNFA group was significantly impaired relative to controls and relative to the AD group on tasks dependent on speech output (including reading, digit span and Stroop inhibition) and verbal semantic knowledge; no other significant differences between the disease groups were identified.

### 3.2 Peripheral hearing

Increasing age was associated with a significant increase in mean response time (detection threshold) at the three highest frequencies tested. Relative to the healthy control group, there was a significant difference (*p*<0.05) in mean detection thresholds for the AD group only at 0.5 kHz (4.1 dB) and 4 kHz (7.2 dB); these threshold elevations were small and unlikely to be clinically relevant, and there were no significant differences at any other frequency tested.

### 3.3 Accent comprehension

**Question comprehension.** One patient with PNFA was unable to understand any questions on the first 20 items of the test (including items presented in the Southern English condition); this patient was excluded from analysis. Group results adjusted for age, gender and education are presented in Table 3. All groups showed a reduction in mean scores for sentences presented in an international accent compared with Southern English. Both patient groups showed a statistically significant reduction in score compared to controls in both international and Southern English accent conditions (*p*<0.01), and the PNFA group performed significantly worse than the AD group in both conditions (*p*<0.05). International minus English difference scores did not differ significantly between any of the groups.

**Word verification.** Within the healthy control group, small but statistically significant differences in score were observed between word verification under international accents compared with the native English accent (see Table 3); details of the control analysis are presented in Supplementary material on-line. Group results adjusted for age, gender and number of years of education, are presented in Table 3. One patient with PNFA performed at chance on all target accents, and was an outlier on target and distractor items in the English accent (obtaining a score of 12/24 on English target items, and a score of 1/24 on English distractor items, where a chance score was 12 for each set). Due to the small patient group size, analyses were conducted excluding this subject's data.

Compared to controls, the AD group showed a significant reduction in score for American and English accents (both *p*<0.05) and there was a trend to worse performance for Australian (*p*=0.07) and South African accents (*p*=0.09), however the mean international accent minus English accent difference score was not significantly different to controls (*p*>0.5). The PNFA group performed significantly worse than controls for American and Australian accents (both *p*<0.05), but not South African (although there was a trend (*p*=0.08) to worse performance). In addition, the PNFA group demonstrated a significantly greater international minus English difference score compared to both control and AD groups (both *p*<0.01).

A qualitative error analysis on match trials for the word verification subtest (raw data presented in Supplementary Table S6 on-line) indicated that patients with PNFA were overall more likely than patients with AD to confuse particular words under international accents, though very few words showed a consistent perceptual cost across international accents.

**Phoneme discrimination.** On this test of phonological processing ability, both patient groups showed a decrease in mean score compared to the control group; however, this difference only attained statistical significance for the AD group (*p*<0.01) (the non-significant PNFA-control difference likely reflects the wide variability in scores for the PNFA group). There was no significant performance difference between the patient groups, although the mean score for the PNFA group was lower than for the AD group.

As the PNFA group showed a decrease in score on this test, group differences in international minus English accent difference score were assessed after adjusting for performance on the phoneme discrimination task by incorporating phoneme discrimination test performance as an additional covariate in the regression model. The adjusted difference between PNFA subjects (*n*=5) and both the AD group (*p*<0.05) and the control group (*p*<0.01) remained significant after adjusting for phoneme discrimination test performance.

**Fig. 1:**
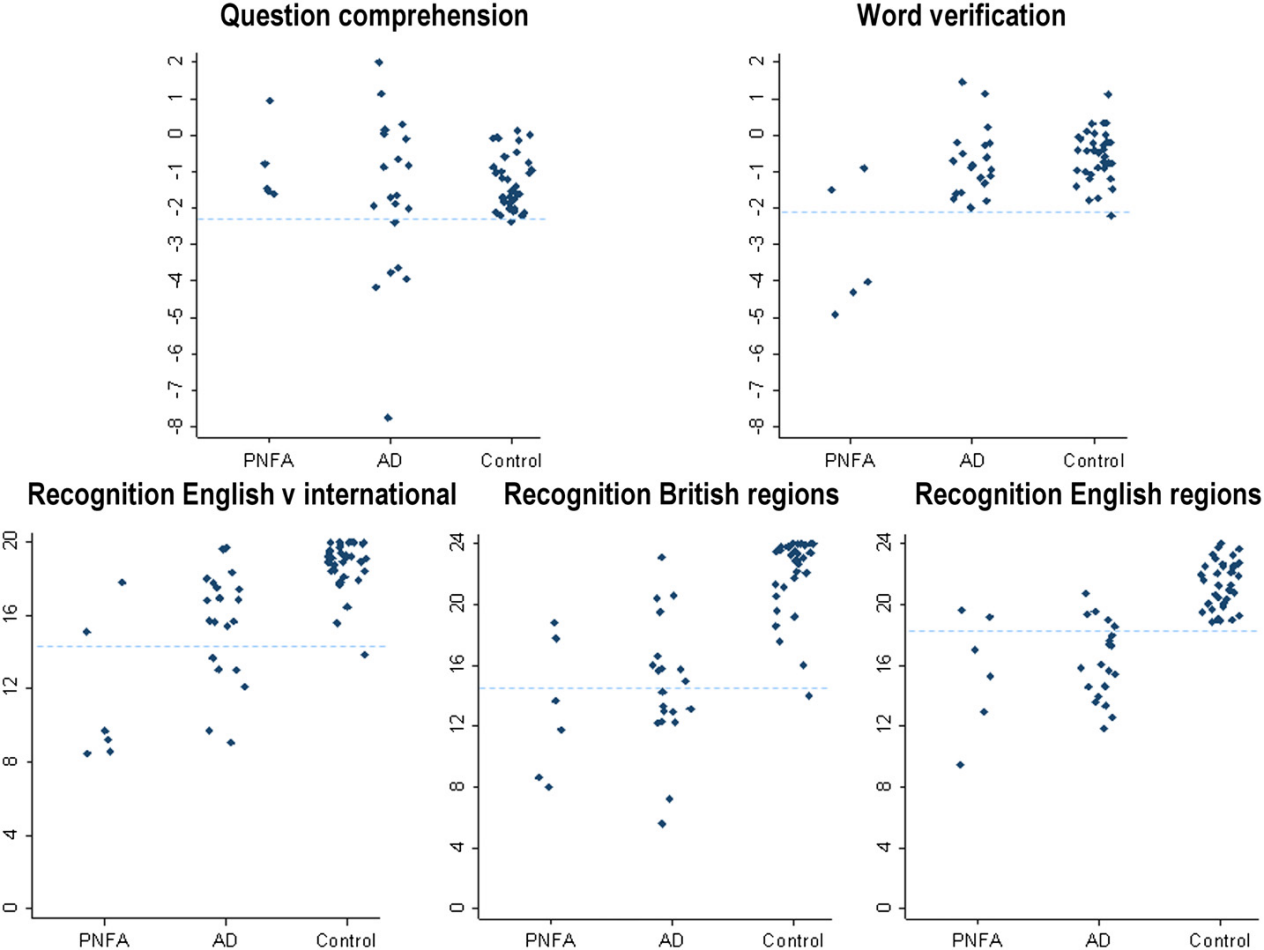
Individual subject data for accent processing performance. Raw data for performance on experimental accent processing tasks are shown for individual subjects in the progressive nonfluent aphasia (PNFA), Alzheimer's disease (AD) and healthy control groups. Accent comprehension data are based on difference scores for question comprehension and word verification in Southern English versus international accents (see text), where a negative score indicates increasing cost for presentation in a non-native accent. Raw scores (/20) for block 1 of the English-versus-international recognition test are shown. Dashed lines represent 5th percentile cut-offs for each subtest calculated from control data. For the English-versus-international accent recognition test, a score of 10 corresponds to chance performance; for the regional British accent recognition test, a score of 6 corresponds to chance performance; for the regional English accent recognition test, a score of 12 corresponds to chance performance.

**Individual patient profiles.** Raw subject data for international minus English difference scores for accent comprehension tests are displayed in Fig. 1. Individual subject performance was classed as impaired if below the 5th percentile cut-off score for the healthy control group. On the question comprehension subtest 5/20 AD patients showed a large performance cost for international accents, falling below the 5th percentile of control values on the international minus English accent difference measure; while no PNFA patients (*n*=5, for whom data were available) performed below the 5th percentile. In contrast, on the word verification subtest 3/5 PNFA patients performed below the 5th percentile of control values on the international minus English accent difference measure, while no AD patients performed below the 5th percentile of control values.

### 3.4 Accent recognition

**International accents.** Two patients with AD and three patients with PNFA performed near or at chance on the first block of this subtest, and therefore did not complete the full 80 item task. Group results adjusted for age, gender and education are presented in Table 3. Both patient groups performed significantly worse than control subjects (*p*<0.01) on both block 1 of this test and the complete set of trials (performed in a reduced set of subjects, as patients at chance on block 1 were excluded) (*p*<0.05). The PNFA group performed worse than the AD group on block 1 of this test (*p*<0.05) and for the complete set of trials, although the latter difference was not statistically significant.

**Regional accents.** A similar profile was found for recognition of British and English regional accents: both patient groups performed significantly worse than controls (*p*<0.01) for recognition of British regional accents, however there was no statistically significant performance difference between the two disease groups.

**Country knowledge.** The AD and PNFA groups performed significantly worse than the control group on all country recognition subtests (*p*<0.05). There was no significant performance difference between the two disease groups. As both patient groups were significantly impaired on tests of country knowledge, differences between the subject groups on accent recognition subtest performance were additionally analysed adjusting for performance on each test of country recognition. Differences between patient groups and controls on accent recognition tests remained significant after adjusting for performance on these tests of general geographical knowledge (*p*<0.05).

**Individual patient profiles.** Raw subject data for accent recognition tests are displayed in Fig. 1. A high proportion of patients in both disease groups (17/20 AD, 4/6 PNFA) performed below the 5th percentile control score on at least one accent recognition subtest. In the PNFA group, the same four patients fell into the impaired range on all three subtests. In contrast, in the AD group, 6/20 subjects were impaired on block 1 of the international versus English accent subtest, 10/20 patients on the regional British accents subtest and 14/20 patients on the regional English accents subtest; only 4/20 patients were impaired on all three subtests.

### 3.5 Neuroanatomical data

**Fig. 2:**
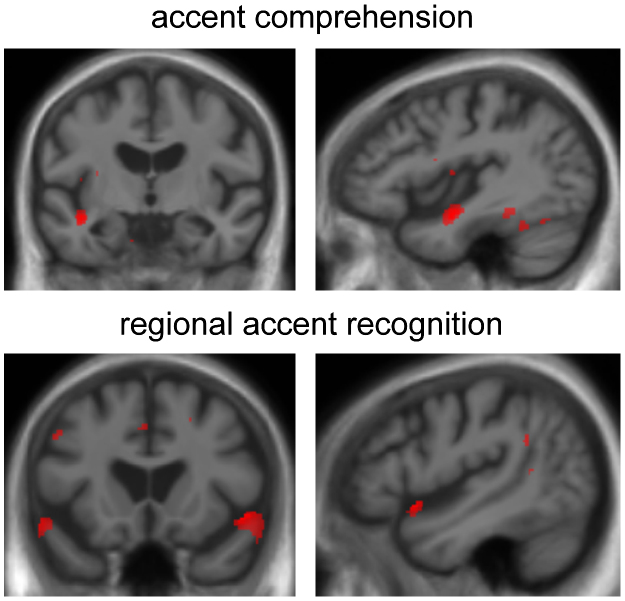
Statistical parametric maps of grey matter volume associated with voice processing performance in the Alzheimer's disease group. Statistical parametric maps (SPMs) show grey matter associations of experimental test performance within the AD group (see also Table 4): upper panels, difference score on question comprehension under international versus Southern English accents (accent comprehension); lower panels, recognition of regional British accents (regional accent recognition). SPMs are presented on sections of the mean normalised T1-weighted structural brain image in DARTEL space. Coronal (left) and sagittal (right) sections are shown. The sagittal sections are derived from the left (upper) and right (lower) hemispheres and the left hemisphere is shown on the left in the coronal sections. SPMs are based on regions for which grey matter associations were significant (*p*<0.05) after correction for multiple comparisons over the pre-specified anatomical small volume (see Table 4); here, SPMs are thresholded at *p*<0.001 uncorrected for display purposes.

Results of the neuroanatomical analyses are summarised in Table 4 and statistical parametric maps are shown in Fig. 2. The AD group showed no significant grey matter associations of experimental test performance after correction for multiple comparisons over the whole brain volume; however, restricting analyses to the pre-specified temporal lobe volumes of interest, the international minus English accent difference score (question comprehension subtest) was positively associated with grey matter in left anterior STG, while performance on the regional British accents recognition subtest was positively associated with grey matter volume in the right anterior STG (*p*<0.05 after FWE correction over the small volume). Additional grey matter associations of performance on each of these subtests were present at an uncorrected significance threshold (*p*<0.001): for the accent comprehension subtest, these areas included left posterior inferior temporal cortex and insula; and for the regional accent recognition subtest, left anterior STG, posterior STS and dorsal prefrontal cortices. No grey matter associations of performance on other accent recognition subtests were identified at the prescribed corrected significance threshold.

## 4 Discussion

Here we have demonstrated impairments of non-native accent comprehension and recognition in patients with two canonical dementias, AD and PNFA. Both patient groups showed impaired recognition of international and regional accents; at the individual subject level, patients with PNFA showed a more consistent pattern of impairment over different (international and regional) levels of accent recognition. The PNFA group showed reduced comprehension of words spoken in international accents compared with a Southern English accent and individual subject data suggested dissociable patterns of impairment: under international accents, patients with AD frequently showed a perceptual cost for comprehension of accented sentences (but not single words), while patients with PNFA frequently showed a perceptual cost for comprehension of accented words (but not sentences). These deficits were not clearly attributable to a general phonological or semantic impairment.

Information about accent processing in neurodegenerative disease is very limited. However, the present findings add to previous evidence for impairments of various aspects of complex auditory pattern processing in these diseases (Goll et al., 2010a,b, 2011, 2012; Hailstone et al., 2011). Such impairments may occur as an early and specific feature of the neurodegenerative process in AD (Gates et al., 2002). More particularly, deficits in the perception and comprehension of prosody have been demonstrated in AD (Allender & Kaszniak, 1989; Horley et al., 2010; Roberts, Ingram, Lamar, & Green, 1996; Taler, Baum, Chertkow, & Saumier, 2008; Testa et al., 2001) and PNFA (Rohrer et al., 2012). As another example of a meta-linguistic vocal signal with segmental, suprasegmental and semantic dimensions, prosody is expected to engage brain mechanisms similar to those involved in accent processing. However, previous studies of nonverbal sound processing in AD and PNFA suggest that these diseases may affect distinct components of vocal signal analysis: whereas AD is predominantly associated with apperceptive deficits of sound pattern analysis under non-canonical listening conditions (Gates et al., 2002; Goll et al., in press), PNFA is predominantly associated with conjoint deficits of timbre and auditory semantic processing suggesting a more fundamental deficit in the encoding of auditory object properties (Goll et al., 2010a, 2011). These core deficits might contribute to the dissimilar patterns of accent comprehension impairment (perceptual cost) shown by individual patients with AD versus PNFA: whereas comprehension of questions is likely to depend on tracking extended auditory patterns, comprehension of monosyllables is more likely to depend on accurate encoding of individual sound objects (here, spoken phonemes). A primary perceptual deficit might lead to degraded representation of accent characteristics and consequently reduced recognition of those accents, or conversely, impaired accent knowledge might damage ‘top-down’ mechanisms that normally act to disambiguate the effects of perceptual distortion. An error analysis here suggested that any perceptual cost associated with presenting phonemes in non-canonical form is likely to represent an interaction of factors which may be partly accent specific. It has been hypothesized that adaptation to unfamiliar accents engages top-down lexically driven categorisation mechanisms (Bradlow & Bent, 2008; Norris, McQueen, & Cutler, 2003); such dynamic mechanisms could plausibly be degraded in neurodegenerative disease and could be assessed in future work. However, the present data do not suggest a clear, consistent perceptual defect across the disease groups, suggesting that additional semantic-level deficits may also play a role in impaired recognition of non-native accents in AD and PNFA. It is plausible a priori that the semantic processing of accents might be aligned with other geographically-organised concepts (Crutch & Warrington, 2003, 2010; Della Rocchetta et al., 1998); however, our data suggest that accent recognition is not merely subsumed by brain mechanisms of geographical semantic processing. The semantic organisation of accents in relation to other kinds of vocal semantic processing remains an open issue.

The neuroanatomical findings in the AD group corroborate these behavioural profiles. A measure of accent comprehension was positively associated with grey matter volume in left anterior STG (though interpretation of this association should be cautious in the absence of a clear overall behavioural cost relative to healthy controls). Recognition of non-native regional accents was positively associated with grey matter volume in a more anterior cortical region in right anterior STG. It is noteworthy that these cortical associations were found within temporal lobe areas somewhat more anterior than those previously implicated in certain other aspects of nonverbal perceptual analysis (Norris et al., 2003; Rohrer et al., 2012) but in close proximity to previously identified cortical associations of voice recognition (Hailstone et al., 2011). This might reflect shared mechanisms for processing the meaning of accents and other dimensions of the speech signal: the processing of accents may depend on brain mechanisms analogous to those mediating speech intelligibility under other forms of perceptual distortion (Binder et al., 2000; Bishop & Miller, 2009; Friederici, Kotz, Scott, & Obleser, 2010; Leff et al., 2009; Scott, Blank, Rosen, and Wise, 2000; Scott et al., 2006; Zatorre, Evans, Meyer, & Gjedde, 1992). Indeed, accented speech could be viewed as an ‘ecological’ example of degraded speech, representing an extreme form of the phonological–phonetic variation exhibited by individual speakers even within the spectrum of a native accent or under varying listening conditions (Best, McRoberts, & Sithole, 1988; Best et al., 2001; Clarke & Garrett, 2004; Flege, Munro, & MacKay, 1995; Floccia et al., 2006; Iverson et al., 2003; Iverson & Kuhl, 2000; Norris et al., 2003; Nygaard & Pisoni, 1998). Comprehension of accented speech may involve assimilation of accented phonemes into categories used for native speech, for example involving matching to stored prelexical templates (Best et al., 1988, 2001; Clarke & Garrett, 2004; Flege et al., 1995; Floccia et al., 2006; Iverson & Kuhl, 2000; Nathan et al., 1998). Tolerance to phonetic variation is likely to be established via exposure to many individual speakers with different accents (Adank et al., 2009; Bradlow & Bent, 2008; Clopper & Bradlow, 2008; Clopper & Pisoni, 2004a; Floccia et al., 2006), the putative template matching algorithm has been shown to be inflexible in infants (Nathan et al., 1998; Schmale & Seidl, 2009) and may be disrupted in neurodegenerative disease. While stored representations of single phonemes are likely to be instantiated in posterior superior temporal cortices (Chang et al., 2010; Jancke et al., 2002; Rauschecker & Scott, 2009; Turkeltaub & Coslett, 2010), decoding of extended utterances such as questions posed in a foreign accent is likely to require tracking of auditory information streams over longer time periods, a function previously localised to more anterior temporal cortices (Friederici, Meyer, & Von Cramon, 2000; Humphries, Willard, Buchsbaum, & Hickok, 2001; Meyer et al., 2002; Scott et al., 2000). A complementary interpretation of the present data in the AD group would hold that accent comprehension depends on stored knowledge about accent properties that also supports accent recognition. Models of speech comprehension and voice recognition (Belin et al., 2004; Belin & Zatorre, 2000; Scott et al., 2006) assign to the anterior STG/STS a key role later in the cortical processing hierarchy for auditory “what” information. It remains to be established how the processing of non-native accents relates to the processing of other vocal properties and how best to incorporate accents in current models of voice recognition.

Our findings suggest that impairments of accent processing may constitute signatures of neurodegenerative diseases and not merely amplification of an effect already present in the normal brain. Healthy control subjects here showed a comprehension performance profile across non-native accents that could reflect past exposure and familiarity with those accents (Adank et al., 2009; Clopper & Bradlow, 2008; Clopper & Pisoni, 2004b) or alternatively, the relative perceptual similarity of the accents chosen here to Southern English (see Supplementary Material on-line), in line with previous suggestions (Clarke & Garrett, 2004; Flege et al., 1995; Floccia et al., 2006; Iverson & Kuhl, 2000; Norris et al., 2003). This normal accent comprehension profile was altered in the patient groups. We argue that the processing of accents is a test case with potentially much broader implications for understanding how the brain encompasses perceptual variation in behaviourally relevant, semantically laden stimuli and how neurodegenerative diseases damage the distributed cortical networks that are presumed to support such processing.

This study has several limitations and suggests a number of directions for future work. Accent processing here was assessed in relation to a limited number of other neuropsychological functions: a more complete understanding of the deficits identified here would require a more detailed investigation of accent processing in parallel with other kinds of complex nonverbal sound processing and a more fine-grained analysis of potentially relevant perceptual and linguistic mechanisms. In this initial study, we set out to sample a broad range of accent processing functions (aspects of accent comprehension and recognition) for accents that were likely to be familiar to our subject population and using various relevant response procedures (sentence comprehension, word verification and forced-choice responses): future work should analyse the component processes in more detail and compare these processes more directly using uniform test procedures. It will be important to assess performance in relation to the specific perceptual characteristics that define particular accents, a key issue in attempting to generalise findings across populations with very different accent exposures. A further dimension is the potential interaction between altered accent perception and distorted production of the patient's own native accent, as illustrated most dramatically in the so-called ‘foreign accent syndrome’ (Hall, Anderson, Filley, Newcombe, & Hughes, 2003; Kurowski, Blumstein, & Alexander, 1996; Luzzi et al., 2008; Van Borsel, Janssens, & Santens, 2005): this would entail a parallel acoustic analysis of patients' spoken output. Even if temporal lobe areas are critical for accent processing, such processing is likely to be mediated by distributed brain networks extending beyond the temporal lobes (Adank et al., 2012; Berman et al., 2003; Burton, Small, & Blumstein, 2000; Peschke, Ziegler, Eisenberger, & Baumgaertner, 2012). A more complete picture of these mechanisms will require complementary functional and connectivity-based imaging techniques, in line with the emerging concept of neurodegenerative diseases as ‘nexopathies’ (Buckner et al., 2009; Seeley et al., 2009; Sonty et al., 2007; Warren et al., 2012). The patient cohorts here were relatively small and assessments were conducted cross-sectionally. This limitation is likely to be particularly relevant to less common and intrinsically heterogeneous syndromes such as PNFA; tests for disease-performance interactions here were likely under-powered. There is a need to address these issues in larger patient cohorts, in other neurodegenerative diseases and longitudinally, in order to establish how accent processing relates to the development of other cognitive deficits and the specificity of deficits for particular neurodegenerative pathologies.

## Figures and Tables

**Table 1:** Summary of demographic and clinical characteristics of patient and control groups.

	**PNFA*****n*****=6**		**AD*****n*****=20**		**Control*****n*****=35**	
	Mean (SD)	Range	Mean (SD)	Range	Mean (SD)	Range
Males: females	0:6	–	8:12	–	13:22	–
Age (years)	66.0 (6.9)	58–76	66.4 (7.6)	49–79	65 (6.0)	54–79
Education (years)	12.3 (3.3)⁎	10–17	13.1 (3.4)⁎	9–20	15.2 (3.3)	11–25
Symptom duration (years)	3.5 (1.3)⁎⁎	2–6	6.0 (2.4)⁎⁎,‡	4–11	n/a	
MMSE score (/30)	20.0 (4.9)⁎⁎	14–26	21.6 (4.1)⁎⁎	14–28	29.4 (0.6)^a^	28–30

MMSE, mini-mental state examination (Folstein, Folstein, & McHugh, 1975); SD, standard deviation.

a *n*=23 controls performed MMSE.

⁎⁎ Significantly lower than controls (*p*<0.01).

‡ Significantly longer than the PNFA group (*p*<0.01).

⁎ Significantly lower than controls (*p*<0.05).

**Table 2:** General neuropsychological assessment in patient and control groups.

	**PNFA*****n*****=6**		**AD*****n*****=20**		**Control*****n*****=35**	
**Test (maxscore)**	Mean (SD)	Range	Mean (SD)	Range	Mean (SD)	Range
**IQ**						
WASI verbal IQ	63.7 (15.1)⁎⁎,‡	55–92	98.3 (16.7)⁎⁎	67–121	120.8 (9.2)	96–142
WASI performance IQ	80.2 (12.1)⁎⁎	70–100	87.9 (16.7)⁎⁎	62–110	116.8 (11.9)	100–141
Reading IQ^a^	78.0 (18.9)⁎⁎,†	56–103	106.4 (16.4)⁎⁎	67–128	118.9 (7.4)	96–129

**Memory**						
Recognition memory (words) (/50)	33.7 (11.7)⁎	19–47	30.7 (7.5)⁎⁎	19–47	47.3 (1.8)	43–49
Recognition memory (faces) (/50)	36.5 (5.4)	30–44	34.9 (5.8)⁎⁎	25–45	42.2 (4.7)	35–49

**Semantic tests**						
BPVS (/150)	127.3 (18.0)⁎⁎	101–146	140.9 (12.4)⁎	106–150	148.1 (1.5)	144–150
GNT (/30)	7.7 (9.5)⁎⁎	0–23	12.1 (8.1)⁎⁎	0–26	26.0 (2.4)	19–30
Synonyms (concrete) (/25)	17.0 (2.9)⁎⁎,†,b	13–20	20.8 (2.7)⁎⁎	13–25	24.3 (1.3)	19–25
Synonyms (abstract) (/25)	17.6 (4.0)⁎⁎,†,b	12–23	20.9 (3.6)⁎⁎	14–25	24.3 (1.2)	20–25

**Working memory**						
Digit span forwards (/12)	4.0 (3.5)⁎⁎,†	0–5	7.5 (2.2)	4–11	8.7 (2.0)	4–12
Digit span backwards (/12)	1.8 (1.7)⁎⁎,‡	1–9	5.2 (2.7)⁎	0–10	7.4 (2.6)	2–12
Spatial span forwards (/12)	4.8 (1.3)⁎,b	4–7	5.7 (2.4)^c^	1–9	6.8 (1.5) d	5–9
Spatial span reverse (/12)	3.8 (2.0)⁎⁎,b	2–6	4.0 (2.0)⁎⁎,c	0–7	6.7 (1.7)^d^	4–10

**Other skills**						
Object decision task (/20)	16.8 (1.9)^b^	14–19	15.7 (2.9)⁎⁎	9–19	18.5 (1.2)	16–20
GDA (/12)	3.0 (3.2)⁎⁎	0–8	5.7 (4.6)⁎⁎	0–14	15.4 (4.8)	6–23
Stroop switching scaled score (/18)	1.2 (0.4)⁎⁎,‡	1–2	3.9 (3.2)⁎⁎	1–11	11.5 (2.0)	7–14

*P* values are for group differences after adjusting for age, gender and years of education. BPVS, British Picture Vocabulary Scale (McCarthy & Warrington, 1992); Concrete and abstract synonyms test (Warrington, McKenna, & Orpwood, 1998); DS, WMS-R digit span tests (Wechsler, 1987); GDA, Graded Difficulty Arithmetic (Jackson & Warrington, 1986); GNT, Graded Naming Test (Warrington, 1997); Object decision task (Warrington & James, 1991); Recognition memory tests (Warrington, 1984); Stroop, D-KEFS Stroop test (Delis, Kaplan, & Kramer, 2001); Visuo-spatial span, WMS-III spatial span (Wechsler, 1999); WASI, Wechsler Abbreviated Scale of Intelligence (Wechsler, 1999).

a Reading IQ measured on the national adult reading test (Nelson, 1982) unless the subject scored ≤15/50 on this test, in which case the Schonell graded word reading test IQ was used (Schonell & Goodacre, 1971).

b *n*=1 PNFA patient did not perform these tasks (different subjects for each).

c *n*=17 AD patients performed this task.

d *n*=8 controls performed this task

⁎ Significantly worse than controls (*p*<0.05).

⁎⁎ Significantly worse than controls (*p*<0.01).

† Significantly worse than AD group (*p*<0.05).

‡ Significantly worse than AD group (*p*<0.01).

**Table 3:** Results for experimental tests in patient and control groups.

		**PNFA*****n*****=6**		**AD*****n*****=20**		**Control*****n*****=35**	
		Mean (SD)	Range	Mean (SD)	Range	Mean (SD)	Range
**Question comprehension**^a^							
English accent	/40	31.4 (6.5)⁎⁎,†	24–40	38.0 (1.4)⁎⁎	35–40	39.3 (0.2)	39–40
International accent	/40	30.4 (6.7)⁎⁎,†	22–38	36.2 (2.2)⁎⁎	31–39	38.6 (0.6)	38–40
Difference score: International—English	/40	−1.0 (1.2)	−2.0, 1.0	−1.8 (2.2)	−8.0, 2.0	−1.3 (0.8)	−2.0, 0

**Word verification**a, b							
English	/24	22.6 (2.6)	18–24	22.6⁎ (2.0)	16–24	23.7 (0.5)	22–24
American	/24	20.0 (3.5)⁎	14–22	21.6 (2.5)⁎⁎	13–24	23.5 (0.7)	22–24
Australian	/24	19.6 (3.5)⁎	15–24	22.1 (2.5)	13–24	23.2 (0.8)	22–24
South African	/24	18.8 (4.7)	13–24	21.7 (1.9)	17–24	22.8 (1.0)	20–24
Difference score: International^c^—English	/24	−2.4 (2.4)⁎⁎,‡	−5.0, 1.0	−0.8 (1.0)	−2.0, 1.3	−0.6 (0.7)	−2.3, 1.3

**Phoneme discrimination**							
Minimal pair word verification	/48	36.7 (13.7)	13–46	42.3 (5.5)⁎⁎	24–47	46.7 (1.1)	44–48

**Accent recognition**							
English versus international (block 1)	/20	11.5 (4.1)⁎⁎,†	8–18	15.7 (3.0)⁎⁎	9–20	18.8 (1.3)	14–20
English versus international (total)^d^	/80	60.7 (10.0)⁎	51–71	64.6 (8.9)⁎⁎	45–75	75.1 (3.2)	64–79
British regions	/24	13.0 (4.8)⁎⁎	7–19	14.9 (4.2)⁎⁎	6–23	22.1 (2.5)	14–24
English regions	/24	15.7 (3.8)⁎⁎	10–20	16.3 (2.5)⁎⁎	12–21	21.3 (1.8)	18–24

**Country knowledge**							
Naming from description	/10	6.7 (2.1)⁎⁎	4–10	7.9 (2.2)⁎⁎	2–10	10.0 (0.2)	9–10
Map naming	/10	5.3 (1.8)⁎⁎	3–8	6.0 (3.1)⁎⁎	1–10	9.4 (0.9)	7–10
Map recognition	/10	7.5 (1.4)⁎	6–10	6.6 (3.4)⁎⁎	0–10	9.9 (0.2)	9–10

Group differences significant after adjusting for background covariates (age, gender, years of education) are displayed.

a Results for *n*=5 PNFA subjects are shown (see text).

b one AD patient declined to continue after three blocks and results were scaled to a score/24 for each accent for this subject.

c Mean score for all three international accents.

d 3 PNFA subjects and 18 AD subjects were able to perform all 80 items on this test.

⁎ Significantly worse than controls (*p*<0.05).

⁎⁎ Significantly worse than controls (*p*<0.01).

† Significantly worse than AD group (*p*<0.05).

‡ Significantly worse than AD group (*p*<0.01).

**Table 4:** VBM data: neuroanatomical associations of experimental test performance in the Alzheimer's disease group.

	**Side**	**Region**	**Z score**	**Cluster size (voxels)**	**MNI Coordinates (mm)**		
**Accent comprehension**							
Difference score: International – English questions	Left	Anterior STG	4.58	130	−42	−8	−18

**Accent recognition**							
British regions	Right	Anterior STG	4.53	171	50	16	−11

Areas listed are based on local maxima exceeding a voxel-wise significance threshold after FWE-correction over the prespecified small volume of interest. All clusters of size >10 voxels are shown. Z scores refer to the local maxima ([x y z], mm) within these regions. MNI, Montreal Neurological Institute; STG, superior temporal gyrus.

## Footnotes

Appendix A Supplementary data associated with this article can be found in the online version at doi:10.1016/j.neuropsychologia.2012.05.027.
