# Supplementary material for: Accent processing in dementia
Source: Neuropsychologia. 2012 Jul;50(9-2):2233–44. doi: 10.1016/j.neuropsychologia.2012.05.027 (PMC3484399; doi:10.1016/j.neuropsychologia.2012.05.027)
Supplement: Supplementary file 1 — Supplementary material [file mmc1.docx]

**SUPPLEMENTARY MATERIAL: Hailstone JC et al., Accent processing in dementia**

**Word verification analyses in healthy controls**

Within the control group, there was strong evidence that word verification scores were lower under all international accents compared with the English accent (mean difference in scores: American: -0.26 (95% CI: -0.46, -0.05); Australian: -0.54 (CI:-0.85, -0.24); South African: -0.89 (CI: -1.25, -0.53), all p<0.0001). Scores for the South African accent were also lower than for the more familiar American accent (difference: -0.64, (CI: -0.96, -0.29); p<0.0001) and for the Australian accent (difference: -0.34, (CI: -0.68, 0.01); p<0.05). Performance was weakly but non-significantly worse for the Australian accent than the American accent (difference: -0.29, (CI: 0.60, 0.03); p=0.08).

The reaction time analysis for items correctly identified showed strong evidence for prolonged mean reaction times (in seconds) for the three international accents compared to the English accent (American: 0.09, (CI: 0.06, 0.11); Australian: 0.11, (CI: 0.08, 0.15); South African: 0.06, (CI: 0.04, 0.09) all p<0.0001). Significant prolonged reaction time was also observed for Australian compared with South African accents (difference: 0.05 (CI: 0.01, 0.09) p<0.05). There was no evidence of differences between reaction times to other international accents (p>0.05).

**Table S1. Characteristics of speakers recorded for the word verification task**

**Speaker No. Nationality Accent Region** Age Time (months) in UK**

1 US General American† Missouri 20 7

2 US General American† North California 20 7

1 Australian General Australian Brisbane 24 9

2 Australian General Australian Melbourne 27 5

1 South African General South African Johannesburg 28 5

2 South African General South African Johannesburg 28 5

1 English Southern English†† Surrey 25 n/a

2 English Southern English†† London 29 n/a

3* English Southern English†† London 43 n/a

4* English Southern English†† Sussex 42 n/a

All speakers were female. *Speakers only in question comprehension task ; **region speakers spent majority of their early life; †speakers’ regions of origin are associated with subtle variation in accent but very similar overall acoustically to General American ; ††this accent is a composite of local variants in London, the Thames Estuary and Home Counties

**Table S2. Spoken sentences in question comprehension test**

|  | **Questions** | **Examples of accepted answers** |
| --- | --- | --- |
| 1 | What is the opposite of young? | old |
| 2 | What is rain made of? | water |
| 3 | What hand do you write with? | right /left |
| 4 | What is the hottest time of the year? | summer/July |
| 5 | What colour is butter? | yellow/golden |
| 6 | What shape is the earth? | round/spherical |
| 7 | How many inches in a foot? | twelve |
| 8 | What is 3 times 10? | Thirty |
| 9 | Can a bird fly? | yes |
| 10 | What does a bell do? | rings/clangs |
| 11 | What is the opposite of good? | bad/evil |
| 12 | What do you sleep in? | bed/pyjamas |
| 13 | What room do you cook in? | kitchen |
| 14 | From what animal do we get milk? | cow |
| 15 | What does a key open? | door/lock |
| 16 | Where do you wear a ring? | finger |
| 17 | What are windows made of? | glass |
| 18 | What is the opposite of white? | black |
| 19 | What colour is the sky? | blue/grey |
| 20 | What do girls grow up to be? | women/ladies |
| 21 | What is the opposite of long? | short |
| 22 | What colour is blood? | red |
| 23 | Where does a picture hang? | wall/gallery |
| 24 | What is a rose? | flower |
| 25 | What's another word for cash? | money/dosh |
| 26 | What does a chicken lay? | egg(s) |
| 27 | What do we hear with? | ears |
| 28 | What are nails made of? | metal/keratin |
| 29 | Who goes to school? | children/pupils |
| 30 | What does an honest man always tell? | truth |
| 31 | What does a watch tell you? | time |
| 32 | What do you burn on an open fire? | wood/coal |
| 33 | How many legs does a dog have? | four |
| 34 | What do you find in a library? | books/computers |
| 35 | What does a bird build? | nest |
| 36 | Who do you see when you're ill? | doctor/nurse |
| 37 | How many pennies in the pound? | 100/240 |
| 38 | What city are we in? | London |
| 39 | What is a very young child called? | baby/ infant |
| 40 | When can you see the moon? | night/evening |

**Table S3. Stimuli used in the word verification task**

|  | **Target** | **Distractor** | **Change** |
| --- | --- | --- | --- |
| 1 | dame | name | 1^st^ Consonant |
| 2 | neat | meat | 1^st^ Consonant |
| 3 | night | might | 1^st^ Consonant |
| 4 | nip | lip | 1^st^ Consonant |
| 5 | pail | tail | 1^st^ Consonant |
| 6 | pill | bill | 1^st^ Consonant |
| 7 | tack | sack | 1^st^ Consonant |
| 8 | bag | back | Last consonant |
| 9 | bean | beam | Last consonant |
| 10 | cab | cap | Last consonant |
| 11 | code | coat | Last consonant |
| 12 | maid | main | Last consonant |
| 13 | bad | bed | Vowel |
| 14 | bat | bit | Vowel |
| 15 | cat | cut | Vowel |
| 16 | deed | dead | Vowel |
| 17 | fall | full | Vowel |
| 18 | gut | get | Vowel |
| 19 | mat | met | Vowel |
| 20 | pit | pet | Vowel |
| 21 | rice | race | Vowel |
| 22 | slap | slip | Vowel |
| 23 | tap | tip | Vowel |
| 24 | tape | type | Vowel |

Word pairs derived from the PALPA Minimal Pairs test

**Table S4. Stimulus trials in the regional accent recognition tests**

|  | **Regional British accents** | |  | **Regional English accents** | |
| --- | --- | --- | --- | --- | --- |
| **Trial** | **Answer** | **Region** |  | **Answer** | **Region** |
| 1 | Wales | Glamorgan | 1 | North | Lancashire |
| 2 | England | Merseyside | 2 | North | Merseyside |
| 3 | Ireland | Londonderry | 3 | South | Essex |
| 4 | Scotland | Scottish borders | 4 | South | Oxfordshire |
| 5 | England | Essex | 5 | South | Hackney |
| 6 | England | Yorkshire | 6 | South | Devon |
| 7 | Scotland | Edinburgh | 7 | North | Lancashire |
| 8 | Wales | Port Talbot | 8 | South | Wiltshire |
| 9 | England | Southern English | 9 | South | Norfolk |
| 10 | Ireland | Limerick | 10 | North | Northumberland |
| 11 | Ireland | Belfast | 11 | South | Gloucestershire |
| 12 | Wales | Glamorgan | 12 | North | Merseyside |
| 13 | Scotland | Glasgow | 13 | North | Yorkshire |
| 14 | Wales | Swansea | 14 | North | Merseyside |
| 15 | Scotland | North Ayrshire | 15 | South | Kent |
| 16 | Ireland | Cork | 16 | North | Yorkshire |
| 17 | Wales | Pembrokeshire | 17 | North | Yorkshire |
| 18 | Ireland | County Antrim | 18 | South | Bristol |
| 19 | Scotland | Glasgow | 19 | North | Durham |
| 20 | England | Southern English | 20 | South | East Sussex |
| 21 | England | Yorkshire | 21 | South | Oxfordshire |
| 22 | Scotland | Aberdeenshire | 22 | North | Cheshire |
| 23 | Wales | Powys | 23 | North | Tyne and Wear |
| 24 | Ireland | Armagh | 24 | South | East Sussex |

**Table S5. Stimuli used in the test of naming of countries from verbal description**

|  | **Country** | **Stimulus** |
| --- | --- | --- |
| 1 | Wales | What country does a leek represent? |
| 2 | Ireland | Which country does Guinness come from? |
| 3 | India | Which country did Ghandi come from? |
| 4 | Germany | Which country did Hitler lead? |
| 5 | Spain | Which country is famous for flamenco and bullfighting? |
| 6 | France | Of what country was De Gaulle the president? |
| 7 | England | In which country are London and Birmingham? |
| 8 | Scotland | From which country does the haggis come? |
| 9 | America/USA | The ‘Stars and Stripes’ is the flag of which country? |
| 10 | Italy | Spaghetti comes from which country? |

**Table S6. Error analysis for word verification task**

| **Word** | **Accent** | | | | | | | | | | | |
| --- | --- | --- | --- | --- | --- | --- | --- | --- | --- | --- | --- | --- |
|  | **Southern English** | | | **American** | | | **Australian** | | | **South African** | | |
|  | AD | PNFA | HC | AD | PNFA | HC | AD | PNFA | HC | AD | PNFA | HC |
| bad |  |  |  | 5 | 20 |  | 15 | 40 |  | 10 | 20 |  |
| bag | 5 |  |  | 5 |  |  |  |  |  | 5 | 20 |  |
| bat |  | 20 |  | 5 |  | 3 | 5 | 40 | 14 | 10 | 20 | 3 |
| bean | 15 | 20 | 3 | 5 | 20 |  |  | 20 |  |  | 60 | 3 |
| cab |  |  |  | 5 |  |  |  |  | 3 | 5 |  |  |
| cat |  | 20 |  | 5 | 40 | 3 |  | 20 |  | 15 | 40 |  |
| code | 5 | 20 |  |  |  |  | 5 | 40 |  |  | 40 |  |
| dame |  |  |  | 15 |  | 6 | 5 |  | 9 |  | 40 |  |
| deed |  |  | 6 | 5 | 20 | 3 | 5 | 20 | 17 | 15 |  |  |
| fall | 5 |  |  | 40 | 100 | 9 | 10 |  |  | 15 |  | 3 |
| gut | 5 |  |  | 5 | 20 |  |  |  | 3 | 20 | 20 | 11 |
| maid |  |  |  | 10 | 20 |  | 5 |  | 6 |  |  |  |
| mat | 5 |  |  |  |  |  | 30 | 80 | 11 |  |  |  |
| neat | 15 | 20 | 3 | 10 | 20 | 6 | 5 | 20 |  | 25 | 20 | 14 |
| night | 5 |  | 3 | 5 | 20 |  | 5 |  | 3 |  |  |  |
| nip |  | 20 |  |  | 60 |  |  |  |  | 10 | 60 | 11 |
| pail | 10 |  |  | 5 |  |  | 10 | 20 |  | 5 |  |  |
| pill | 5 |  |  | 10 |  | 3 |  |  | 3 | 45 | 40 | 66 |
| pit | 10 |  | 6 | 10 |  |  | 5 | 20 |  | 5 | 20 |  |
| rice |  |  |  | 15 | 20 | 6 | 25 | 20 | 14 | 10 | 20 |  |
| slap | 15 |  |  | 15 |  |  |  |  |  | 5 |  | 3 |
| tack |  |  | 3 |  |  | 3 |  | 40 |  | 5 | 40 | 6 |
| tap | 5 | 20 | 6 | 5 | 20 |  | 15 | 60 | 3 | 5 | 20 |  |
| tape | 15 |  |  | 20 | 20 | 9 |  |  |  | 5 | 40 | 3 |

The Table shows proportion of subjects (% of subjects assessed, rounded to nearest) in each group making confusion errors under each accent on ‘match’ trials for each of the words in the word verification subtest. Blank cells indicate no subjects made errors on that word. AD, Alzheimer’s disease; HC, healthy controls; PNFA, progressive nonfluent aphasia
